# Measuring TiO_2_N and AgHEC Airborne Particle Density during a Spray Coating Process

**DOI:** 10.3390/toxics10090498

**Published:** 2022-08-27

**Authors:** Sara Trabucco, Antti Joonas Koivisto, Fabrizio Ravegnani, Simona Ortelli, Ilaria Zanoni, Magda Blosi, Anna Luisa Costa, Franco Belosi

**Affiliations:** 1 CNR-ISAC, Institute of Atmospheric Sciences and Climate-National Research Council of Italy, Via Gobetti 101, 40129 Bologna, Italy; 2 Air Pollution Management APM, Mattilanmäki 38, 33610 Tampere, Finland; 3 CNR-ISTEC, Institute of Science and Technology for Ceramics-National Research Council of Italy, Via Granarolo 64, 48018 Faenza, Italy

**Keywords:** effective particle density, particle emission, spray coating

## Abstract

Effective particle density is a key parameter for assessing inhalation exposure of engineered NPs in occupational environments. In this paper, particle density measurements were carried out using two different techniques: one based on the ratio between mass and volumetric particle concentrations; the other one based on the ratio between aerodynamic and geometric particle diameter. These different approaches were applied to both field- and laboratory-scale atomization processes where the two target NPs (N-doped TiO_2_, TiO_2_N and AgNPs capped with a quaternized hydroxyethylcellulose, AgHEC) were generated. Spray tests using TiO_2_N were observed to release more and bigger particles than tests with AgHEC, as indicated by the measured particle mass concentrations and volumes. Our findings give an effective density of TiO_2_N particle to be in a similar range between field and laboratory measurements (1.8 ± 0.5 g/cm^3^); while AgHEC particle density showed wide variations (3.0 ± 0.5 g/cm^3^ and 1.2 + 0.1 g/cm^3^ for field and laboratory campaigns, respectively). This finding leads to speculation regarding the composition of particles emitted because atomized particle fragments may contain different Ag-to-HEC ratios, leading to different density values. A further uncertainty factor is probably related to low process emissions, making the subtraction of background concentrations from AgHEC process emissions unreliable.

## 1. Introduction

The advent of nanotechnology and the increasingly widespread industrial application of nanomaterials have led to rapidly growing human and ecosystem exposure to NanoParticles (NPs). Determining the potential hazards of NPs is therefore essential to avert potential health hazards while reaping the benefits of nano-enabled products. Worker safety, consumer health and environmental protection concerns require appropriate metrics for the evaluation of NPs’ safety during production, handling, use and recycling. NPs are released into the environment by primary sources such as natural phenomena, combustion processes and industrial activities but also during the generation and handling of engineered NPs. The amount of NPs present in the inhaled air is typically referred to as the exposure level. However, in terms of human health, the more relevant measure is the biological dose, i.e., the number of particles reaching the lung epithelium whose effects can be determined on the basis of toxicological dose–response measurements using either in vivo (animal) or in vitro (cell) models. Inhaled airborne particles are mainly deposited on the lung epithelium by diffusion, sedimentation and impaction [1].

Particle deposition modeling in the human lung is a truly multidisciplinary task, requiring information about physics, biology and mathematics. Biological expertise entails knowledge of lung morphology and the cylindrical geometry of the airways, but also of respiratory parameters determining air flows and particle velocities in the airstream. The physics involved includes the fluid dynamics of an inhaled air volume, i.e., airflow patterns in the lung, particle properties and their deposition mechanisms in order to calculate deposition fractions in specific airway segments [2]. An important key to accurate in vitro dosimetry is the characterization of sedimentation and diffusion rates of nanoparticles suspended in culture media, which largely depend upon the effective density and diameter of formed agglomerates in suspension. An accurate determination of the effective particle density can determine the rate of NPs deposition and thus the delivered dose in an in vitro system. Effective particle density can differ from raw material density and depends on pores distribution and volume, it will influence NPs’ mass concentration. Difficulties in ascertaining particle density lead to uncertainties about the quantification of material deposition and the area of the lung affected.

Effective density is therefore a key parameter in assessing inhalation exposure of engineered NPs in an occupational environment. When assessing the impact of engineered NPs on human health, a distinction should be made between incidental and background engineered NP concentrations, which usually require time-resolved particle size distribution measurements [3]. However, although sufficiently sensitive, particle mass distribution measurements available online are not technically feasible at the time resolutions occurring in industrial processes involving engineered NP synthesis and formulation. In addition, online size-resolved monitoring techniques rely mainly on mobility, aerodynamic and optical diameter measurements, which provide particle number concentrations [4]. Although particle number concentration discriminates between background concentrations and process emissions, it does not provide reliable information for risk assessment since a human health risk is typically characterized by mass concentration. However, particle number size distributions can be translated to the mass distribution when effective particle density is known. This approach was successfully demonstrated in numerous occupational exposure assessment studies [5,6,7,8,9].

To investigate the pulmonary toxicity of various atmospheric aerosols or airborne engineered nanomaterials, cell-based in vitro exposure systems are adopted as alternatives to in vivo studies using laboratory animals. The air–liquid interface (ALI) exposure protocol is suitable for insoluble particles such as engineered nanomaterials, overcoming the difficulty to control the exposure dose when these particles are floating in the culture medium and the cells are placed at the bottom of the culture dish (submerged culture system). Although technically more difficult, the ALI cell exposure system is a realistic airborne particle exposure method, since in vivo exposure to these particles occurs at the air–liquid interface when inhaled particles come into contact with organs such as the lungs [10]. The quartz crystal microbalance technique was proposed as an accurate and sensitive real-time direct monitoring method of the cell-delivered aerosol dose in air–liquid interface cell culture experiments [11]. The number of particles reaching the cells located at the bottom of the multi-well plate is governed by particle kinetics (settling, diffusion and agglomeration phenomena). Changes in their surface charge/chemistry over time alter particle characteristics, inter-particles interactions and the manner in which they are transported to cells. Not only affected by the particle properties (size, density, and surface chemistry) these processes are also impacted by the solution properties (viscosity, density, presence of proteins or co-exposed agents, etc.) [12].

Accurate dosimetry, therefore, requires accurate characterization of suspended particles, in particular, effective diameter and density, which, along with the density and viscosity of the suspending fluid, determine mass transport phenomena. In the case of liquid-suspended engineered nano-enabled materials (ENM) the sedimentation coefficient can be measured directly by analytical ultracentrifugation (AUC), thereby eliminating the need to explicitly determine effective density [13], or by combining particle effective mass obtained by centrifugal field-flow fractionation and by particle size obtained by transmission electron microscopy (TEM) [14]. However, the air-to-liquid phase change of nano-suspended materials through atomization processes might give rise to changes in the ENM and the formation of agglomerates. While ALI exposure experiments involve cloud aerosol particles generation using vibrating mesh generators, with a count median diameter (CMD) of a few microns [15], the spray coating process in industrial pilot plants, which is a considerable potential worker risk source and requires the need for deeper investigation to assess risk [16], determines higher droplet diameter (as explained below). Archer et al. (2020) showed different droplet nuclei morphologies, and therefore density values, depending on droplet evaporation kinetics [17]. Low density or hollow particles are advantageous for several applications, and especially for pulmonary drug delivery where they improve dispersibility and delivery efficiency by lowering the aerodynamic diameter of the particles. Several particle design strategies were developed to create voids in particles and lower their density by changing their Peclet number [18,19].

The European Research Project Anticipating Safety Issues at the design stage of NAno product development (ASINA, H2020-GA 862444) aims to support the industrial uptake of nanotechnology by providing Safe by Design (SbD) solutions and supporting tools to broaden awareness and understanding among entrepreneurs of SbD’s potential in important areas such as the nano design feature of coating and encapsulation. The project comprises a pilot phase involving test beds, pilot plants and case studies to test and validate the methodology contents as specific implementations that can be generalized to other nanomaterials and industrial case studies. The ASINA project includes field campaigns to assess the emission of NPs from lab-scale and industrial-scale spray coating processes in the production of antimicrobial/self-purifying polyester and plastic surfaces. This paper presents different methods to determine the particle densities of two target NPs (N-doped TiO_2_, TiO_2_N and AgNPs capped with a quaternized hydroxyethyl-cellulose, AgHEC), generated in both industrial-scale and lab-scale spraying processes.

TiO_2_ and AgNPs were identified as target materials because of their industrial scale relevance [20,21,22,23] as antimicrobial/self-purifying additives for different kinds of products as investigated within the ASINA project.

## 2. Materials and Methods

ENM Materials. Two NP suspensions were used in the spray nozzle pilot plant: N-doped TiO_2_, TiO_2_N (1% *w/w*) dispersed in ethanol (solution 96% grade solvents, VMR international) and AgNPs capped with a quaternized hydroxyethylcellulose, AgHEC, (Dow Chemical, Midland, MI, USA) dispersed in water at concentrations of 0.1%, 0.05% and 0.01% *w/w*. Specifically, the TiO_2_N suspension was prepared by Colorobbia Italia, SPA (Sovigliana Vinci, FI, Italy) while the AgHEC aqueous nano suspensions were produced by CNR-ISTEC (Faenza, Italy) using a patented production process [24]. TiO_2_N suspension density was 0.824 g/cm^3^ considering a bulk TiO_2_ density of 4.23 g/cm^3^ (Rutile form) at 1% *w/w* concentration. Primary TiO_2_N particle size was 80.0 ± 0.2 nm obtained by Dynamic Light Scattering (DLS) measurements.

The AgHEC suspended in water at concentrations of 0.1%, 0.05% and 0.01% *w/w* (zeta potential 17.5 ± 1.5 mV) had density of 1.013 g/cm^3^, 1.006 g/cm^3^ and 1.001 g/cm^3^ respectively, considering a bulk Ag density of 10.5 g/cm^3^. The hydrodynamic diameter of the AgHEC particles, including the polymeric shell adsorbed on the surface, was 273 nm (DLS), while TEM analysis showed an Ag primary particle size of 17.8 ± 2.1 nm (see Figure S1, Supplementary Material). The AgHEC bulk particle density, by considering the insoluble components as derived both from nominal composition or Inductively Coupled Plasma-Optical Emission Spettroscopy (ICP-OES) analysis (see Supplementary Material, Tables S1 and S2) was around 1.4 g/cm^3^.

Particle density measurements were carried out using two different approaches: one based on the ratio between mass and volumetric particle concentrations obtained during field measurements or with laboratory-generated particles; the other based on the ratio between aerodynamic and geometric particle diameter as measured by an inertial particle spectrometer.

(I) Mass-to-volume ratio: data from field measurements at the industrial pilot plant and on the laboratory scale

Field measurements were conducted at an industrial spray coating pilot plant near Florence (Wiva Group now Witek) belonging to the ASINA project consortium. Detailed descriptions of the industrial process, atomized suspensions and measurement protocols used during the field campaign (February 2021) are given in our previous paper [25]. Briefly, the automatic spray coating machine is conveyor belt-operated, the substrate passing through a plasma neutralizer to the spray chamber and then to a drying oven. The spray chamber volume is about 6 m^3^ with an inflow rate of about 3000 m^3^/h clean air and a bottom aspiration flow in order to maintain pressure conditions inside the chamber. No forced ventilation is present in the working area. Figure S2 shows SEM pictures of TiO_2_N and AgHEC particles collected inside the spray chamber during spray coatings. Since the process is continuous, the cabin cannot be completely sealed because of the entrance and exit openings for the conveyor belt. A plasma neutralizer is optionally used to impart a negative charge to the surface of polymethyl methacrylate panels (PMMA) and promote adhesion of the coating to the substrate. Spraying is entirely automated, the spray nozzles moving over the substrate, and carried out inside a ventilated chamber. The four nozzles of each sprayer can be operated singly, in pairs or concomitantly. The spray nozzle (manufacturer and model are confidential) operated with 270 L/min air flow, atomizing the coating suspension delivered at a flow rate of 200 mL/min per nozzle. After spraying, the substrate is dried in a drying oven.

Aerosol concentration measurements were carried out to characterize emissions into the environment from PMMA and textile substrates during the process. Size resolved particle number and mass concentrations were obtained at Near Field (NF) position after the spray chamber at heights from 1 to 1.3 m. The real-time NF particle measurement equipment included, among others, an SMPS (Grimm L-DMA and Grimm CPC mod. 5403, Grimm Aerosol Technik, Ainring, Germany), an OPC (Grimm mod 11 D Grimm Aerosol Technik, Ainring, Germany) and an aerosol photometer (DustTrack II mod. 8530, TSI Inc., Shoreview, MN, USA). All instruments were calibrated by the manufacturer prior to the campaign. Particle size distributions obtained by SMPS and OPC were merged by averaging the number of countings in overlapping size bins between SMPS and OPC. Mobility and optical particle diameters were assumed to be the same. The SMPS used a soft X-ray neutralizer (mod. 3087, TSI Inc., Shoreview, MN, USA) and the particle countings were corrected using the transfer function obtained by Nicosia et al. (2018) [26]. The diffusion losses along the SMPS sampling tube were corrected according to Gormerly and Kennedy (1948) [27]. Off-line gravimetric particle mass (PM) samples (total fraction) were taken simultaneously at NF by collecting particles on filters (PTFE, 1 µm porosity) at 50 L/min flow rate (Bravo H-Plus, TCR Tecora, Cogliate, Italy) and weighing the filters on a 5 digit analytical balance (Mettler, Toledo AX105). Each test session comprised 4 sprays and lasted about 40 min. Figure S3 shows a typical time series of particle number concentrations measured at NF with TiO_2_N spray.

**Figure 1:**
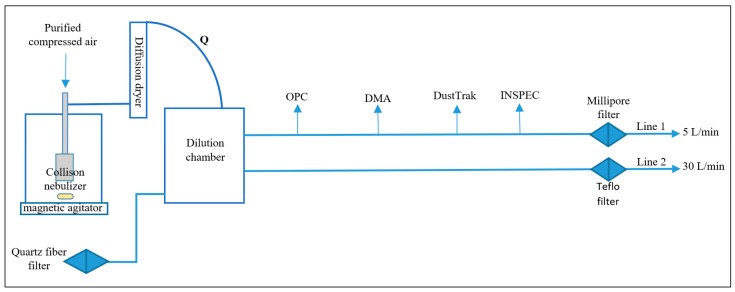
Diagram of experimental setup. A magnetic agitator was used to maintain uniform suspension composition, given the high bulk material densities.

**Figure 2:**
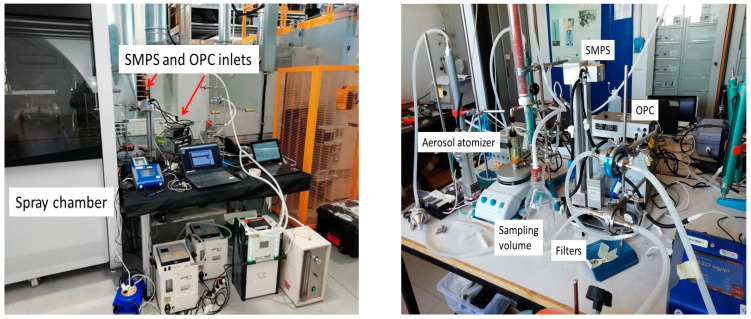
Experimental field campaign setup. On the left the setup used at field campaigns; while on the right at the laboratory.

The laboratory tests were carried out with a Collison-type nebulizer (BGI Inc., Cambridge, MA, USA) working at 1 bar air pressure to atomize both the TiO_2_N and AgHEC suspensions. Figure 1 shows a scheme of the experimental setup. The atomized droplets pass through a silica gel column leaving a dry residual, which is then sent to a one-liter mixing volume. Two parallel sampling lines were connected to the aspiration inlet of two pumps (Dual Bravo, XearPro, Cogliate, Italy). One line sampled the aerosol on an absolute PTFE filter for gravimetric assessment (flow rate 1800 L/h), while the other one was dedicated to real-time simultaneous sampling aerosol instruments: OPC (Grimm, mod. 11 D, Ainring, Germany), SPMS (Grimm, L-DMA, Ainring, Germany) and an aerosol photometer (DustTrack II mod. 8530, TSI). A magnetic agitator (Velp Scientifica srl, Usmate, Italy) was deployed to maintain uniform suspension composition. Figure 2 shows a picture of the field and laboratory apparatus. To maintain the particle number concentrations within the SMPS and OPC upper counting levels, the TiO_2_N suspension was diluted in ethanol by a factor of 100 until 0.0001% *w/w* concentration (zeta potential 124.0 ± 0.6 mV) and the AgHEC suspension diluted in MilliQ water by a factor of 10 until 0.01% and 0.005% *w/w* concentrations.

(II) Direct single-particle density measurement: Inertial Spectrometer (INSPEC)

The INertial SPECtrometer [28,29] allows for measuring the particle aerodynamic diameter. It consists of a rectangular duct with a 90° bend through which clean air flows (Figure S6). The channel at the bend is 1.9 mm deep and 20 mm wide. A thin aerosol sheath is injected upstream of the bend and the overall flow is sucked downstream through an acetate cellulose or polycarbonate filter membrane. The aerosol is sampled at a 7 L/h flow rate and injected into a sheet clean air flow of 360 L/h. The air velocity inside the INSPEC vertical channel is laminar. Due to the 90° bend, the particles are separated according to their size leaving the original streamlines by a distance that is a unique function of their inertia and resistance forces. The particles remain airborne until they deposit on the filter, being magnified by geometric projection on the filter surface. The inertial spectrometer was calibrated in terms of aerodynamic size as a function of deposition distance by making use of monodisperse and polydisperse aerosols (see Supplemental Material, Figures S7 and S8). INSPEC was deployed at the NF location and during the laboratory-generated aerosols. A polycarbonate filter (Nuclepore, porosity 0.2 µm, Whatman) was used as particle deposition surface. Strips were cut at different deposition distances at known aerodynamic particle diameters, while observations with electronic microscope (FESEM, carl Zeiss Sigma NTS, Gmbh Öberkochen, Germany) allowed the determination of the geometric particle diameter at the same deposition distance. Single particle density can be obtained by using the following relation [30]:

(1) $$\rho_{p}=\sqrt{\frac{d_{A}}{d_{G}}}$$

where ρ_p_ is the single particle density value; d_A_ is the particle aerodynamic diameter; and d_G_ is the particle geometric diameter. In the above equation, the dynamic form factors of the particles were considered unitary.

Before starting the coating process at the field campaign, background concentrations were obtained to determine volume aerosol size distributions, gravimetric particle mass (PM) and photometer mass concentrations. The values obtained were considered as the blank condition to be subtracted from the values obtained during the TiO_2_N and AgHEC spray coating processes. As filter particle mass concentration assessment requires large sampling volumes, it was not possible to obtain a filter for each spray test. In fact, only one filter each for the TiO_2_N and AgHEC coatings was obtained (see Table S3 for test descriptions). The aerosol photometer allows real-time particle mass concentrations based on particle light scattering. It responds linearly to the aerosol mass concentration only for particle diameters similar to the wavelength of the incident light and the scattered light depends also on the particle refractive index, a complex number including both the absorptive and scattering component, and shape. Furthermore, all optical instruments are influenced by relative humidity, which tends to increase the output sensor signal and, therefore, the aerosol mass concentration [31,32]. It follows that photometer data must be corrected by a factor given by the ratio between gravimetric and real-time measurements obtained simultaneously. Provided the aerosol particle composition does not change too much, this corrected factor can be used to obtain gravimetrically corrected particle mass concentrations.

AgHEC and TiO_2_N particles generated in the laboratory can be influenced by MilliQ water and ethanol impurities, respectively. Incomplete water evaporation inside the silica gel column can also influence aerosol size distribution. As MilliQ water contains several impurities [33,34], it was nebulized alone and the aerosol volume size distribution subtracted from the atomization sprays containing AgHEC. The same procedure was applied to the TiO_2_N sprays subtracting the particle number concentrations obtained by atomizing ethanol alone (Figures S4 and S5).

Uncertainties were calculated by considering the variability of the volume aerosol size distribution and filter weighing procedure. The first uncertainty factor may be very large in the case of field measurements on account of the temporal variability of the sources (spray coating is an intermittent process). The second factor of uncertainty was estimated by weighing a set of blank filters.

## 3. Results

### 3.1. Particle Density by Mass to Volume Ratio: Industrial Pilot Plant

Table 1 reports the density values determined by the mass-to-volume ratio, using data from field measurements at the industrial pilot plant. The PM concentrations were obtained with gravimetric filter weighing and aerosol particle volumes by the aerosol size distributions at NF (assuming that all particles were spherical in shape). The background concentration obtained on 15 February 2021 was subtracted from the TiO_2_N and AgHEC spray concentrations.

**Figure 3:**
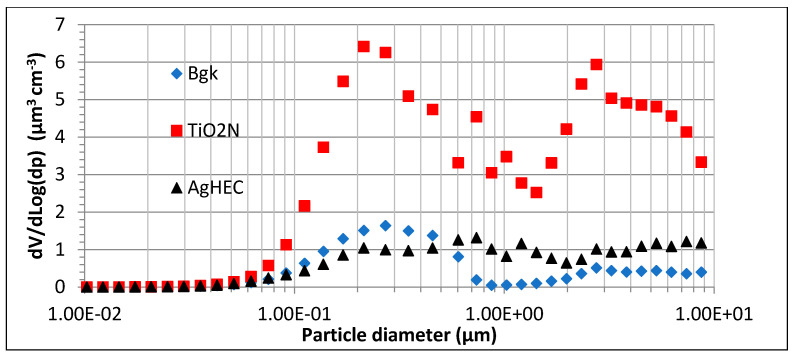
Averaged volume particle size distributions for background (Bgk), TiO_2_N and AgHEC at field campaigns.

Spray tests using TiO_2_N were observed to release more and bigger particles than tests with AgHEC, as indicated by the particle mass concentrations and volumes measured on the filters. In addition, given the intermittent source term, particle volume size distribution variability is very high. We concluded that a time-variable source term makes the method insufficiently accurate to obtain reliable effective particle density values. This is not only due to the high concentration variability but also to the long sampling time required to determine gravimetric particle mass concentrations, which in turn obliges us to work with time-averaged particle volume size distributions that may be comparable to the background in the case of low particle emissions. Figure 3 gives the volume particle number concentrations obtained during the field campaigns in a semi-log scale graph for background, for the TiO_2_N and AgHEC sprays, respectively.

The volume size distributions are bimodal with the first mode around 0.3–0.5 µm, showing comparable volume contributions for background and AgHEC. The second mode is around 2–3 µm more visible for TiO_2_N than AgHEC particles.

### 3.2. Particle Density by Mass-to-Volume Ratio: Lab-Scale Atomizer

**Figure 4:**
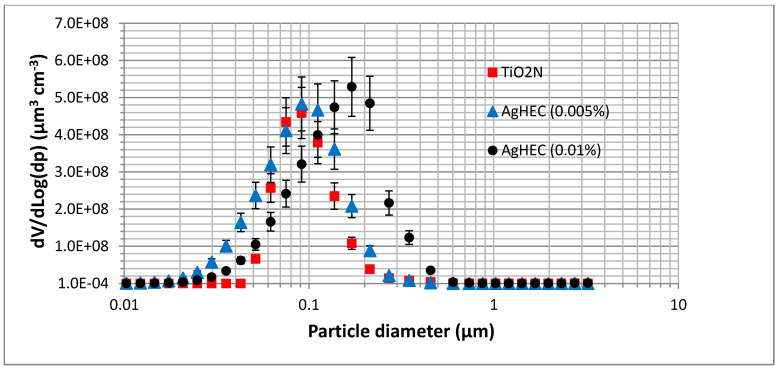
TiO_2_N and AgHEC volume size distributions obtained with laboratory-generated suspensions. Bars show one standard deviation.

Figure 4 shows the volume size distributions obtained by the laboratory-generated aerosols: TiO_2_N (0.0001%) and AgHEC at two different concentrations (0.01% and 0.005%). The volume size distribution of the water and ethanol solvents were removed. Volume size distributions peaked at around 80/90 nm and at about 200 nm for the more concentrated AgHEC suspension.

The differences between the aerosol volume size distributions obtained at the industrial pilot plant scale and in the laboratory may be due to the different atomization processes or to dilution used in the laboratory tests to maintain particle number concentrations within the instrument counting level ranges. The spray atomizer used at Witek gives rise to an average droplet size of around 20/30 µm (Sauter mean diameter), and a droplet residue of a few microns: 1–2 µm for AgHEC, and about 3–4 µm for TiO_2_N (see Supplementary Material for details), fairly similar to the field monitoring findings. The Collison-type atomizer used for the laboratory tests gives rise to a droplet volume median diameter of 2.5–3 µm at 1 bar working pressure [35], one order of magnitude lower than the droplet diameter from the air blast atomizer at the Witek pilot plant.

Averaged total particle volume concentration for TiO_2_N atomization was 164 ± 50 µm^3^/cm^3^, the mass concentration measured on the filter 358 ± 17 µg/m^3^, and the calculated effective particle density 2.2 ± 0.8 g/cm^3^.

Averaged total particle volume concentration for AgHEC atomization was 249 ± 76 µm^3^/cm^3^, the mass concentration measured on the filter 313 ± 68 µg/m^3^, and the calculated effective particle density 1.3 ± 0.7 g/cm^3^. In this case, the measured density is comparable to the theoretical bulk density obtained by considering a large amount of insoluble ingredients (hydroxyethyl cellulose matrix) whose presence could delay the evaporation of liquid, avoiding the formation of voids.

Data provided by the OPC (Figure 3 shows most of the particles to be in the OPC counting range) and the aerosol photometer allow the analysis of particle concentration only during spraying activity, obtaining better discrimination with respect to the background. Prior to this, however, a calibration factor must be introduced for the aerosol photometer. The ratio between aerosol photometer and gravimetric particle mass concentration at background measurement was 1.5. Table 2 gives the corrected particle mass concentrations obtained by the aerosol photometer, the volume particle concentration obtained with the OPC and the effective particle density values for TiO_2_N tests. Table 3 shows the concentration obtained for AgHEC. All the data were background subtracted using the particle concentrations before and after each spray (all measurements being carried out at 1 s^−1^ or 0.18 s^−1^ frequency for the aerosol photometer and OPC, respectively). Uncertainties in particle effective density values were below 30%.

The average density for TiO_2_N particles was calculated to be 2.0 ± 0.7 g/cm^3^. Sprays onto PMMA surfaces were shown to give rise to more reproducible particle density values than sprays on textiles.

Averaged density fro AgHEC particles was calculated to be 3.7 ± 0.6 gr/cm^3^.

### 3.3. Direct Particle Density Measurements (INSPEC): Pilot Plant and Laboratory Scale

During the spray coating campaign in Witek, a filter was sampled by means of INSPEC at the NF position for each nanomaterial under investigation (TiO_2_N, AgHEC, see Figure S9). Table 4 shows three sections of the INSPEC deposition filter, corresponding to three aerodynamic diameters. By measuring particle geometric diameters and considering the deposition distance, the aerodynamic diameter, and as consequence, the density of the particle can be obtained from the instrument calibration curve with an uncertainty of about 10%. Averaged densities resulted in about 1.7 g/cm^3^, and 1.2 g/cm^3^ for TiO_2_N and AgHEC, respectively. However, smaller and larger particles were also observed in the AgHEC filter at the same deposition distances, indicating for the smaller particles a density of around 4.2 g/cm^3^, and for the larger particles a density of around 0.6 g/cm^3^. This may suggest that some HEC residuals might be separated from the Ag material giving rise to much higher or lower particle density values if some residual of separated Ag or HEC were collected.

Nevertheless, in the laboratory tests, most of the particles were observed to depart from inertial particle behavior (<0.5 µm aerodynamic size), and their density values could not be obtained.

Table 5 gives a summary of the particle density values obtained by means of the different atomization processes and measurement techniques for both particle suspensions.

**Figure 5:**
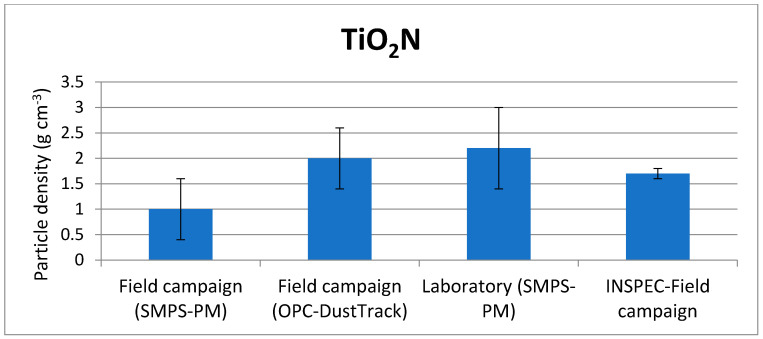
Titanium dioxide atomized particle density values obtained with different techniques and atomization processes.

TiO_2_N: The particle density values obtained with the different techniques align with the experimental error (see Figure 5). The average particle density was 1.8 ± 0.5 g/cm^3^ (or 2.0 ± 0.1 g/cm^−3^, excluding data of the “Field campaign (SMPS-PM)”, half the theoretical bulk density, as expected considering the porous structure of atomized granules.

**Figure 6:**
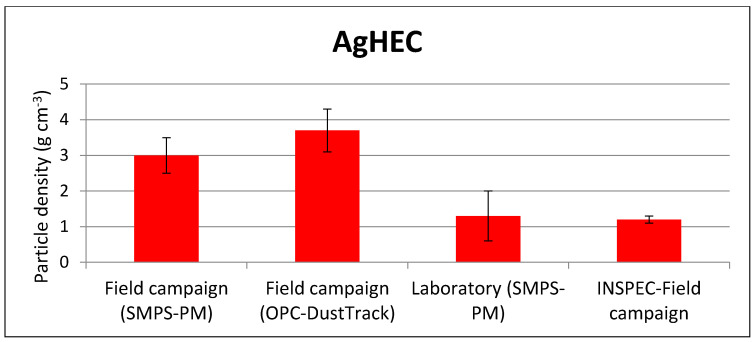
AgHEC atomized particle density values obtained with different techniques and atomization processes.

AgHEC: article density values were found to differ with various measurement techniques and between different atomization processes (see Figure 6). In general, we observed two different particle density values: approximately 3/3.5 g/cm^3^ and 1.2 g/cm^3^. This finding leads to speculation regarding which particle remains as a composite unit, once the suspension was atomized. Particle fragments may also contain different Ag-to-HEC compositions, leading to different density values. SEM observations and further tests should be carried out to investigate this aspect.

## 4. Discussion

AgHEC and TiO_2_N NPs emitted in a spray coating process at an industrial scale were characterized by real-time samplers and offline techniques. The same materials were nebulized during a laboratory-scale investigation using a Collison nebulizer (Venturi type atomizer). Different particle morphologies, aggregation conditions, and therefore effective particle density values were calculated for the two different aerosol generation techniques.

The effective particle density values found during field measurements are usually challenged citing varying background concentrations and the non-continuous process that makes it difficult to obtain repeatable measurements. The effective densities calculated by Koivisto et al. (2022) for TiO_2_N and AgHEC were 2.1 and 6.5 g/cm^3^, respectively [9]. Their calculation was based on previous day gravimetric mass measurement, considered as the background mass concentration, as well as on online particle mobility, optical size-resolved particle number concentration, and gravimetric mass measured during the process. While the effective density of TiO_2_N was found to be in a similar range to our own findings (Figure 5), AgHEC particle density was overestimated ca. 1.8 times compared to our SMPS-PM and OPC-DustTrack field measurements, and ca. 5 times compared to our laboratory SMPS-PM and INSPEC field measurements. Table 6 gives the cumulative particle mass concentration below 1 µm and below 4 µm (respirable fraction) obtained with the different effective particle densities and by considering the averaged volume size distributions obtained during the field monitoring campaign.

The particle mass concentration scales linearly with the effective particle density value. Therefore in the case of titanium oxide particles, the particle mass concentrations belonging to the respirable fraction are comparable, while for AgHEC particles the differences are more relevant.

Wide variations in AgHEC particle densities in the field and in the laboratory assessments are probably related to low process emissions, making the subtraction of background concentrations from AgHEC process emissions unreliable. A further uncertainty factor consists in the assumption that process emissions in the coating suspensions consist only of NPs. While this assumption is a justified precautionary consideration, it may not reflect the NP density if other particles are emitted as well. Thus, the effective particle density measured in situ may be case-specific to the measurement conditions and cannot be applied generally. It follows that effective particle density determinations should be conducted under laboratory conditions where steady-state conditions can be guaranteed and background particles are not present. Nevertheless, for this laboratory-based measurement to be applied to the field campaign, the atomization process used in the laboratory should be representative of the field one.

Alternatively, a combination of instruments providing high time resolution concentrations based on geometric and aerodynamic particle diameters, like, for example, an OPC and an Aerodynamic Particle Sizer, could contribute to providing reliable effective particle density values in the presence of unsteady NP sources in concentrations comparable with the background.

## 5. Conclusions

This work emphasizes the importance of measuring the effective density of airborne particles. In order to determine this key parameter, a multi-criteria approach was adopted. The use of two different methods applied at both an industrial-scale pilot plant and in the laboratory implies taking into account a huge amount of variables. Our findings give an effective density of TiO_2_N particle to be in a similar range between field and laboratory measurements, while AgHEC particle density showed wide variations. We hypothesized that these variations are related to low process emissions, making the subtraction of background concentrations from AgHEC process emissions unreliable. In addition, a possible fragmentation of the AgHEC composite during the spray process, with the formation of particles containing different Ag-to-HEC compositions can justify different density values. Finally, a further uncertainty factor consists in the assumption that process emissions in the coating suspensions consist only of NPs. While this assumption is a justified precautionary consideration, it may not reflect the NP density if other particles are emitted as well. Overall, the multi-method approach put in place to obtain an accurate measurement of aerosolized particle density evidenced how the values are affected by the atomization methods, the techniques used to calculate density and the instruments used to measure particles emission, even if for homogeneous phases such as TiO_2_-N the densities measured with different approaches are within the experimental error.

## Figures and Tables

**Table 1:** Results of the field campaigns. Background subtracted.

Spray	PM	ΣniVi	ρ
	Gravimetric	(µm^3^/cm^3^)	(g/cm^3^)
	(µg/m^3^)		
Background	28 ± 1	15 ± 6	1.9 ± 0.8
(15/02/2021)			
TiO_2_N^1^	92 ± 1	91 ± 50	1.0 ± 0.6
(Test 1–6)			
AgHEC ^1^	36 ± 1	12 ± 2	3.0 ± 0.5
(Test 7–13)			

^1^ See Table S3 for test characteristics.

**Table 2:** TiO_2_N sprays. Aerosol mass concentrations were determined by aerosol photometer while total particle volume was obtained by the OPC. All data refer exclusively to spray spikes. The aerosol photometer data were corrected by the gravimetric correction factor.

Spray	Aerosol	ΣniVi	ρ
(TiO_2_N)	Photometer	(µm^3^/cm^3^)	(g/cm^3^)
	(µg/m^3^)		
Test 1 (200 mL/min-PMMA)	38	22	1.7
Test 2 (400 mL/min-PMMA)	47	31	1.6
Test 3 (800 mL/min-PMMA)	212	123	1.7
Test 4 (200 mL/min-Textile)	49	17	2.9
Test 5 (400 mL/min-Textile)	100	38	2.6
Test 6 (800mL/min-Textile)	162	124	1.3

**Table 3:** AgHEC sprays. Aerosol mass concentrations were determined by aerosol photometer, while total particle volume was obtained with the OPC. All the data refer exclusively to spray spikes. The aerosol photometer data were corrected by the gravimetric correction factor.

Spray	Aerosol	ΣniVi	ρ
(AgHEC)	Photometer	(µm^3^/cm^3^)	(g/cm^3^)
	(µg/m^3^)		
Test 7 (200 mL/min-0.01% Textile)	10.0	2.5	4.0
Test 8 (400 mL/min-0.01% Textile)	16.0	3.6	4.4
Test 9 (200 mL/min-0.05% Textile)	18.6	5.8	3.2
Test 10 (400 mL/min-0.05% Textile)	30.3	9.5	3.2

**Table 4:** Direct particle densities measured at the Witek monitoring campaign by INSPEC.

Deposition Section mm	Aerodynamic Diameter (µm)	TiO_2_-N	Ag-HEC
		Experimental Density (g/cm^3^)	Experimental Density (g/cm^3^)
Witek field campaign			
23–30	3.3	1.5	1.1
30–42	2.1	1.6	1.2
42–48	1.3	2.1	1.2
Averaged density (g/cm^3^)		1.7 ± 0.3	1.2 ± 0.1

**Table 5:** TiO_2_N and AgHEC particle densities obtained with different measurement techniques and different atomization processes. Bulk: theoretical raw material density; Agglomeration: highest packing density (Keplero conjecture); Field campaign (SMPS-PM): density values obtained considering the particle mass collected on filters and volumetric aerosol size distribution at the field campaigns; Field campaign (OPC-DustTrack): particle density obtained using only OPC particle volumetric size distribution data and real-time mass particle concentration values from DustTrack at field campaigns Laboratory (SMPS-PM): the same as Field campaign (SPMS-PM) but with laboratory-generated aerosols; Direct measurement—Field campaign (INSPEC): single particle density measurement obtained with filters sampled with INSPEC in field campaign.

Suspension	TiO_2_N	AgHEC
	Density (g/cm^3^)	Density (g/cm^3^)
Bulk	4.2	1.4
Agglomeration (Keplero cong.)	3.1	1.0
Field campaign (SMPS-PM)	1.0 ± 0.6	3.0 ± 0.5
Field campaign (OPC-DustTrack)	2.0 ± 0.6	3.7 ± 0.6
Laboratory (SMPS-PM)	2.2 ± 0.8	1.3 ± 0.7
Direct measurement—Field campaign (INSPEC)	1.7 ± 0.1	1.2 ± 0.1

**Table 6:** TiO_2_N and AgHEC particle mass concentrations below 1 µm and 4 µm by taking into account the measured averaged volume size distribution during the field campaign and different particle density values: present work and Koivisto et al. (2022), [9].

Particle Density	TiO_2_N		AgHEC	
(g cm^−3^)	(µg m^−3^)		(µg m^−3^)	
	<1 µm	<4 µm	<1 µm	<4 µm
1.8 (present work)	71	129		
2.1 [9]	83	150		
3 (present work)			10	25
6.5 [9]			22	54

## Data Availability

The data are available on reasonable request from the corresponding author.

## Supplementary Materials

The following are available online at https://www.mdpi.com/article/10.3390/toxics10090498/s1, Table S1: Theoretical AgHEC particle density obtained from the suspension composition, Table S2: Experimental bulk AgHEC particle density obtained with ICP-OES analysis, Table S3: Test description, Table S4: Aerodynamic diameter and uncertainties as a function of the deposition distance, Figure S1: TEM micropicture of Ag nanoparticles. Figure S2: Above: TiO_2_N particles. Bottom: AgHEC particle. Particles sampled inside the spray chamber, Figure S3: Left: a scheme of a test made of four sprays. Right: time series of particle number concentration measured with OPC at NF position. The spikes are well visible, Figure S4: Volume particle size distributions of TiO_2_N nebulized suspension and Ethanol, Figure S5: Volume particle size distributions of AgHEC (0.01%) and AgHEC (0.0005%) nebulized suspensions and MilliQ water alone, Figure S6: INSPEC working principle, Figure S7: INSPEC experimental calibration curve, Figure S8: PLS particles at two different deposition distances. Left: 26 mm. Right: 36 mm. Figure S9: Aerosol particles sampled by means of the INSPEC during field measurements and deposited at 45 + 3 mm distance (1.4 µm aerodynamic diameter from Figure S7). Left: AgHEC particles, from test 10-11-12 (Table S3); right: TiO_2_N particles, from test 1-2-3 (Table S3). References [25,36,37,38,39,40] are cited in the supplementary materials.
