# Supplementary material for: Measuring TiO2N and AgHEC Airborne Particle Density during a Spray Coating Process
Source: Toxics. 2022 Aug 27;10(9):498. doi: 10.3390/toxics10090498 (PMC9503037; doi:10.3390/toxics10090498)
Supplement: Supplementary file 1 [file toxics-10-00498-s001.zip › toxics-1870505-supplementary.pdf]

toxics

MDPI

MEASURING TiO_2N AND AgHEC AIRBORNE PARTICLE DENSITY DURING A SPRAY COATING PROCESS

Sara Trabucco [1], Antti Joonas Koivisto [2], Fabrizio Ravegnani [1], Simona Ortelli [3], Ilaria Zanoni [3], Magda Blosi [3], Anna Luisa Costa [3] and Franco Belosi [1,*]

1. Nano Engineered Materials (NEM)

TiO_2N suspension: 0.1% in ethanol. Suspension density of 0.824 gr cm^{-3} considering a raw TiO_2 density of 4.23 gr cm^{-3} (Rutile form). Primary particle size determined by Dynamic Light Scattering (DLS): 80 nm.

AgHEC suspensions: 0.1%, 0.05% and 0.01% w/w in water. Averaged suspension densities: 1.013 g/cm^3, 1.006 g/cm^3, and 1.001 g/cm^3 respectively, considering a raw Ag density of 10.5 g/cm^3. Primary AgHEC particle size: 273 nm (DLS) (while at TEM, Ag particle size detected was of 17.8 $\pm$ 2.1 nm, see Figure S1).

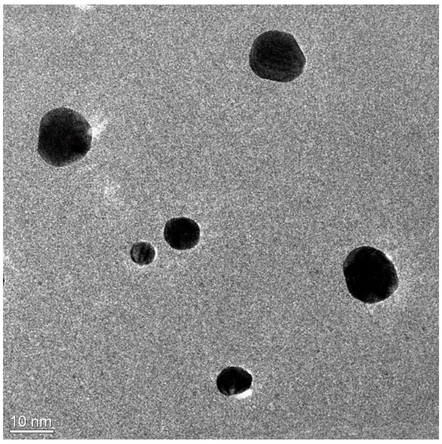

Figure S1. TEM micropicture of Ag nanoparticles.

Table S1 shows the theoretical particle density obtained from the suspension composition. Table S2 gives the experimental bulk density obtained by means of ICP-OES analysis on coated pieces (collected during the experimental campaign).

Figure S2 shows SEM pictures of TiO_2N and AgHEC particles collected inside the spray chamber during spray coatings. As expected, the forced flow of the suspension through the nozzle combined with solvent evaporation induces agglomeration of nanoparticles and cellulose into spherical units of a much larger sizes than values obtained at DLS.

The Keplero conjecture is a mathematical theorem about sphere packing in three-dimensional Euclidean space. It states that no arrangement of equally sized spheres filling space has a greater average density than cubic close packing (face-centered cubic) and hexagonal close packing arrangements. The density of these arrangements is around 74.05% [1]. This implies a maximum packing density of 3.13 g/cm^3 and 1.04 g/cm^3 for TiO_2N and AgHEC, respectively.

Table S1. Bulk AgHEC particle density calculated by considering nominal suspension composition.

Composition	Material percentage (%)	Raw Density (g/cm^3)	AgHEC density
Ag	8.6	10.5	
HEC (Hydroxyethylcellulose)	78.5	0.36	1.5 g/cm^3
NaCl	12.9	2.13	

Table S2. Bulk AgHEC particle density calculated by considering ICP-OES composition.

Composition	Material percentag e (%)	Raw Density (g/cm^3)	AgHEC density
Ag	7.2	10.5	
HEC (Hydroxyethylcellulose)	77.4	0.36	1.4 g/cm^3
NaCl	15.4	2.13	

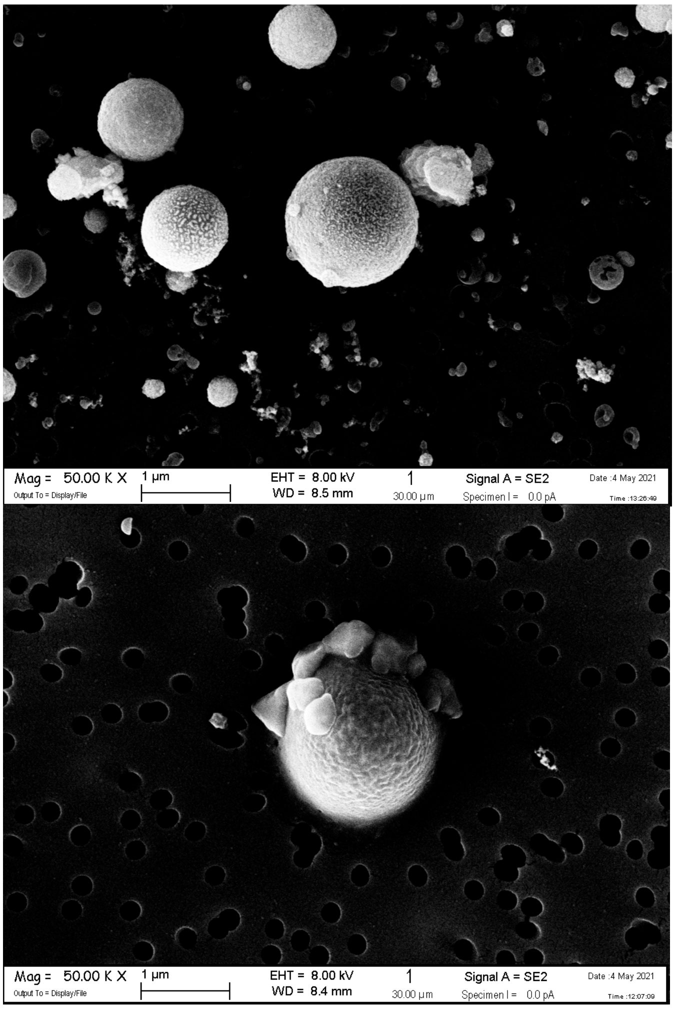

Figure S2. Above: TiO_2N particles. Bottom: AgHEC particle. Particles sampled inside the spray chamber.

2. Field campaign test description

Each test session comprised 4 sprays and lasted about 40 minutes. Fig. S3 shows a typical time series of particle number concentrations measured at NF with TiO_2N sprays.

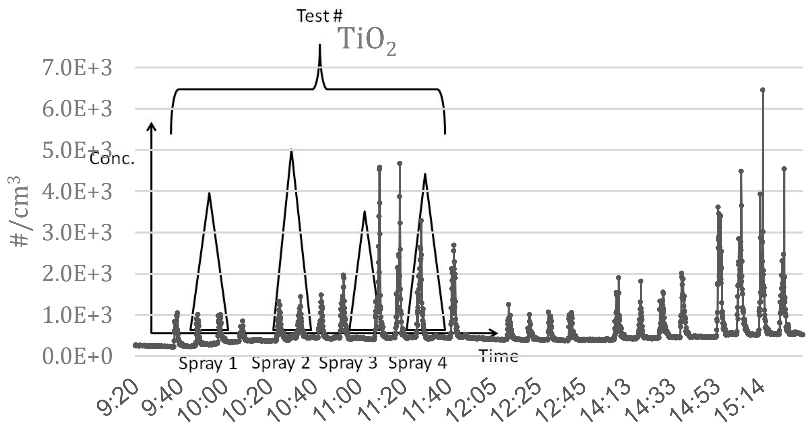

Figure S3. Left: a scheme of a test made of 4 sprays. Right: time series of particle number concentration measured with OPC at NF position. The spikes are well visible

Table S3. shows the suspension used, the flow rate and the substrate of each test carried out (for more details see [2]).

Table S3. Test description.

Test	Material	Nozzle	Flow rate (mL/min)	Substrate	Spray time
1	TiO_2N	1	200	PMMA	6'15"
2	TiO_2N	2	400	PMMA	7'33"
3	TiO_2N	4	800	PMMA	9'38"
4	TiO_2N	1	200	Textile	6'18"
5	TiO_2N	2	400	Textile	7'49"
6	TiO_2N	4	800	Textile	10'02"
7	AgHEC (0.01%)	1	200	Textile	6'10"
8	AgHEC (0.01%)	2	400	Textile	6'52"
9	AgHEC (0.05%)	1	200	Textile	5'46"
10	AgHEC (0.05%)	2	400	Textile	6'57"

3. Laboratory aerosol generation

Figure S4 and S5 show the volume aerosol size distribution for TiO_2N and AgHEC laboratory generated aerosols, respectively. The graphs also report the Ethanol and MilliQ residuals.

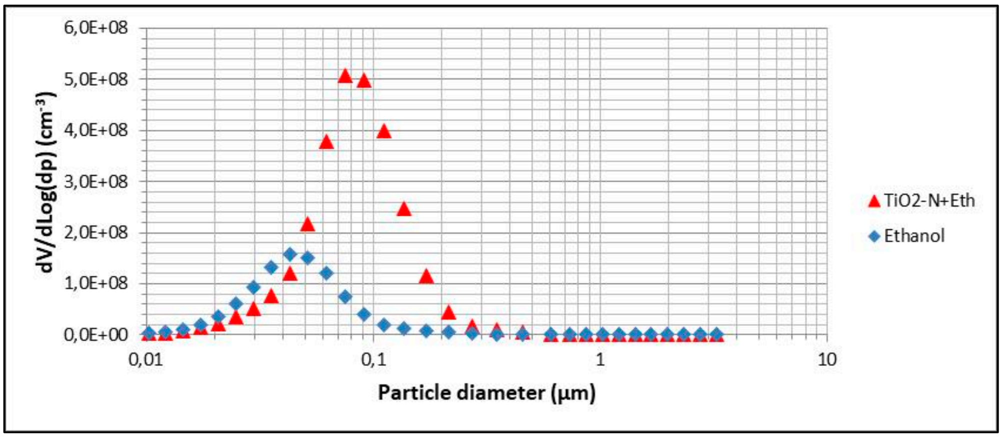

Figure S4. Volume particle size distributions of TiO_2N nebulized suspension and Ethanol.

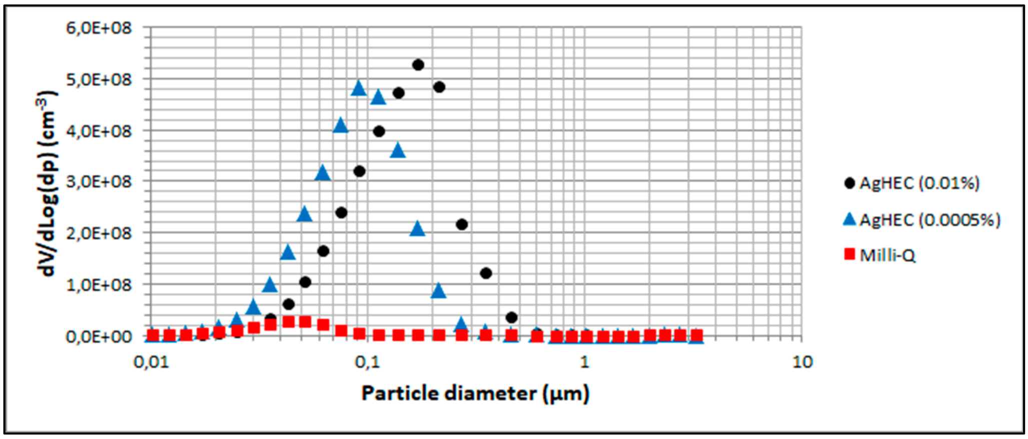

Figure S5. Volume particle size distributions of AgHEC (0.01%) and AgHEC (0.0005%) nebulized suspensions and MilliQ water alone.

3. INSPEC working principle and calibration

INertial particle SPECtrometer consists of a rectangular duct with a 90° bend through which clean air flows (Figure S6). The channel at the bend is 1.9 mm deep and 20 mm wide. A thin aerosol sheath is injected upstream of the bend and the overall flow is sucked downstream through a wall of the duct made of an acetate cellulose or polycarbonate filter membranes. The aerosol is sampled at 7 L/h flow rate and injected into a sheet flow of 360 L/h. The air velocity inside the INSPEC vertical channel is laminar. Due to the 90° bend the particles are separated according to their size, leaving the original streamlines by a distance which is a unique function of their inertia and resistance forces [3]. The particles remain airborne until they deposit on the filter, being magnified by aerodynamic projection to the filter surface. The useful filter area is a rectangle 54 mm long and 20 mm wide

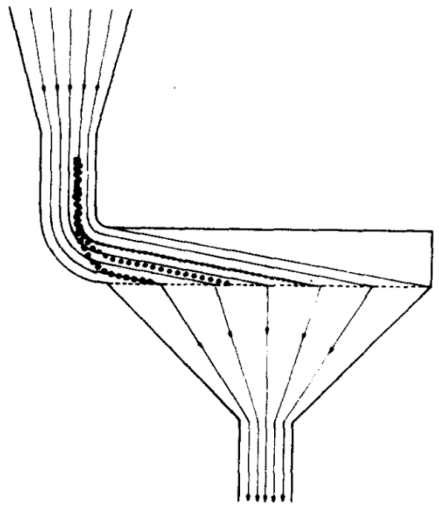

Figure S6. INSPEC working principle.

The inertial spectrometer was calibrated by means of monodisperse and polydisperse aerosols.

Monodisperse particles. Two suspensions were prepared, each one with 500 μl of 1 and 2 μm of PSL standard (Duke Standards, Thermo Fisher Scientific, Waltham MA, USA) diluted respectively in 30 and 40 ml of MilliQ water. The aerosol droplets were generated by using a Collison nebulizer at 1 bar pressurized air (BGI, Inc.). The droplets went through a silica gel column, where the water was removed, after which they were sampled by the INSPEC for 5 minutes.

Polydisperse particles. Polystyrene dissolved in Xilene (C8H10) was used to obtain spherical polydisperse particles with density equal to 1 g/cm^3 (the geometric diameter coincides with the aerodynamic diameter). The suspension was then atomized by means of a medical nebulizer. Particle deposition distances were measured by optical and SEM microscope (SEM, Table Top Hitachi 3000). Figure S7 shows the deposition distance (calibration curve) as a function of the particle aerodynamic diameter for all experimental conditions and aerosol types.

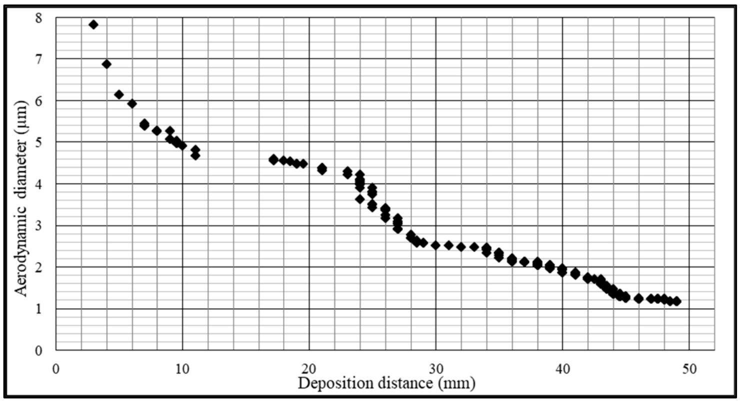

Figure S7. INSPEC experimental calibration curve.

The calibration curve in the particle aerodynamic diameter range of 7-2 μm shows a linear dimensional separation as a function of the deposition distance. At larger or lesser deposition distances the calibration curve has a non-linear trend, meaning that the inertial separation is weaker. The final section of the calibration curve is influenced by the low inertial effects of small particles. On the opposite site, the calibration curve also has another non-linear section due to the effect of the boundary layer inside the bend, which reduces the flow velocity and therefore the Stokes number responsible for particle separation. Figure S8 shows an example of PSL particles observed at two different deposition distances, while Figure S9 shows an example of AgHEC (left) and TiO2N (right) particles sampled at 45+3 mm deposition distance.

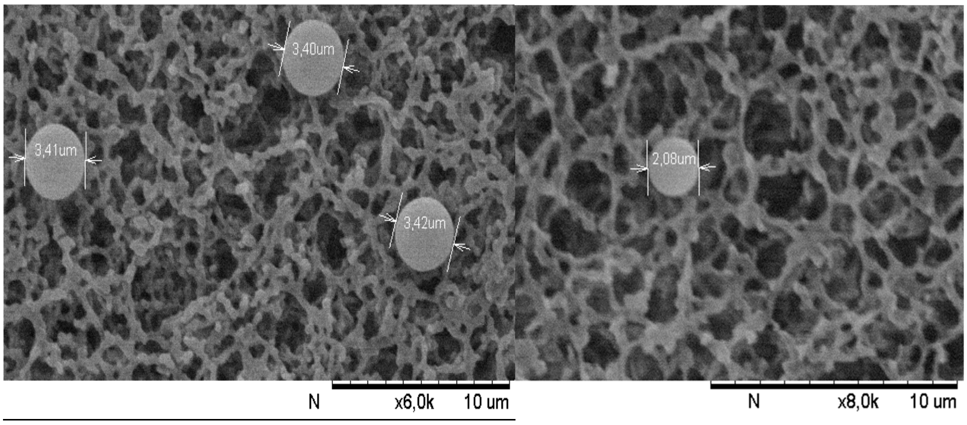

Figure S8. PLS particles at two different deposition distances. Left: 26 mm. Right: 36 mm.

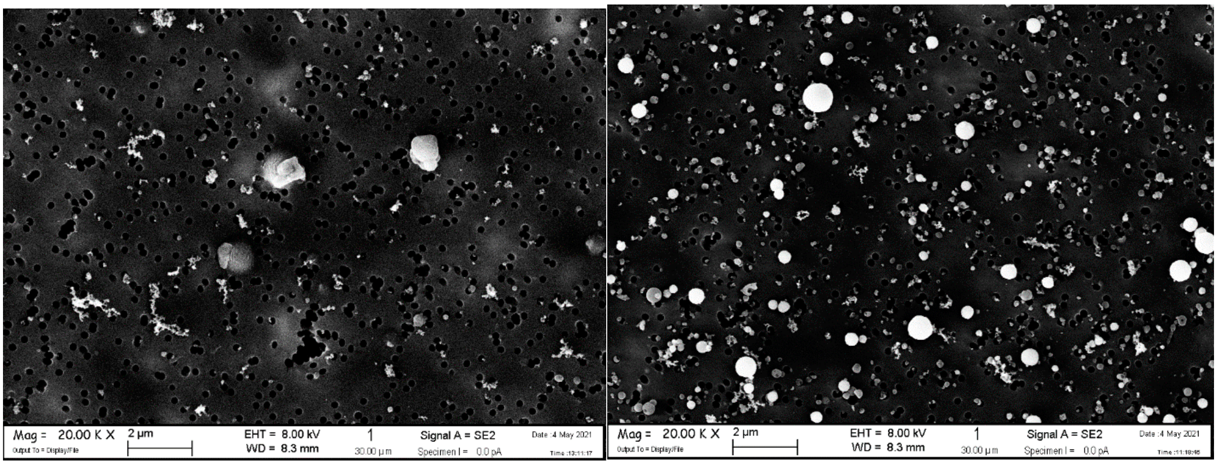

Figure S9. Aerosol particles sampled by means of the INSPEC during field measurements and deposited at 45 $\pm$ 3 mm distance (1.4 μm aerodynamic diameter from Figure S7). Left: AgHEC particles, from test 10-11-12 (table S3); right: TiO_2N particles, from test 1-2-3 (table S3).

Table S4 shows the particle average aerodynamic diameter at four different deposition distances. Uncertainties is about 10% for particle sizes larger than 3 μm. For smaller particle diameters, the inertial effects are lower and the instrument's separation capability is reduced. Therefore, only deposition distances smaller than 40 mm will be considered.

Table S4. Aerodynamic diameter and uncertainties as a function of the deposition distance.

Deposition distance (mm)	26±3	36±6	45±3
Averaged aerodynamic diameter (μm)	3.4±0.5	2.1±0.2	1.4±0.2

3. Spray Droplet Volume

An often described equation for predicting averaged droplet sizes produced by pneumatic atomization is [4]:

$$SMD=\frac{585\ 10^3}{U_R}\left(\frac{\sigma_L}{\rho_L}\right)^{0.5}+1683\ \mu_L^{0.45}\sigma_L{\rho_L}^{-0.225}\left(\frac{1000}{J}\right)^{1.5} \quad \text{(S1)}$$

Where:

SMD is the Sauter mean diameter (μm), ϱL the liquid density (kg/m³), σL the liquid surface tension (N/m), UR the velocity of air relative to the liquid at the nozzle exit (m/s), μL the liquid viscosity (Pa s) and the air-to-liquid volume ratio (dimensionless). The liquid and air flow rates used during the sprays were 255 cm³/min and 270 L/min, respectively. Using the above equation, the calculated SDM values were 24 μm and 32 μm for AgHEC (water solvent), and TiO_2N (ethanol solvent), respectively. These values are in agreement with the individual droplet size distributions of Ye et al. (2017) at 220 L/min air flow rate (comparable with the air flow rate used in our tests) [4]. After complete water or ethanol evaporation, droplet nuclei will be between 1-2 μm for AgHEC (according to the different suspension concentrations) and about 3 μm for TiO_2N suspension. The steady-state evaporation of a water droplet of diameter, d, is proportional to the surface area of the droplet. Evaporation rate, k, is defined as

$$d^2\,(t) = d_0^2 - kt \quad \text{(S2)}$$

where d(t) is the droplet diameter at time t and d_0 is the initial droplet diameter. Evaporation rate coefficients for pure water droplets at different air temperature are given by Vehring et al.(2017) in the spray drying process [5]. At ambient temperature, a k value of 1.2 μm²/ms may be considered. A droplet with an initial diameter of 24 μm will decrease in size down to few microns in half a second. The same order of magnitude was given by Lieber et al (2021) [6] when investigating the evaporation rate of saliva droplets. Ethanol solvent will evaporate faster. Since the time scale between spray generation and aerosol measurement at NF was about 1 minute, we can assume complete droplet evaporation i.e., droplet nuclei being the mainly particle composition, sampled at NF.

References

1. Hales, T.C. An Overview of the Kepler Conjecture. *arXiv Mathematics e-prints* **1998**, math/9811071.
2. Del Secco, B.; Trabucco, S.; Ravegnani, F.; Koivisto, A.J.; Zanoni, I.; Blosi, M.; Ortelli, S.; Altin, M.; Bartolini, G.; Costa, A.L.; et al. Particles Emission from an Industrial Spray Coating Process Using Nano-Materials. *Nanomaterials* **2022**, *12*, 313, doi:10.3390/nano12030313.
3. Mueller, R.; Kleinebudde, P. Comparison of a Laboratory and a Production Coating Spray Gun with Respect to Scale-Up. *AAPS PharmSciTech* **2007**, *8*, E21–E31, doi:10.1208/pt0801003.
4. Ye, Q.; Pulli, K. Numerical and Experimental Investigation on the Spray Coating Process Using a Pneumatic Atomizer: Influences of Operating Conditions and Target Geometries. *Coatings* **2017**, *7*, 13, doi:10.3390/coatings7010013.
5. Vehring, R.; Foss, W.R.; Lechuga-Ballesteros, D. Particle Formation in Spray Drying. *Journal of Aerosol Science* **2007**, *38*, 728–746, doi:10.1016/j.jaerosci.2007.04.005.
6. Lieber, C.; Melekidis, S.; Koch, R.; Bauer, H.-J. Insights into the Evaporation Characteristics of Saliva Droplets and Aerosols: Levitation Experiments and Numerical Modeling. *Journal of Aerosol Science* **2021**, *154*, 105760, doi:10.1016/j.jaerosci.2021.105760.
